# Integrative analysis of fitness and metabolic effects of plasmids in *Pseudomonas aeruginosa* PAO1

**DOI:** 10.1038/s41396-018-0224-8

**Published:** 2018-08-10

**Authors:** Alvaro San Millan, Macarena Toll-Riera, Qin Qi, Alex Betts, Richard J. Hopkinson, James McCullagh, R. Craig MacLean

**Affiliations:** 1 Department of Zoology, University of Oxford, Oxford, OX2 6GG UK; 2 Department of Microbiology, Hospital Universitario Ramon y Cajal (IRYCIS) and Network Research Centre for Epidemiology and Public Health (CIBERESP), 28034 Madrid, Spain; 3 Department of Evolutionary Biology and Environmental Studies, University of Zurich, CH-8057 Zurich, Switzerland; 4 Swiss Institute of Bioinformatics, Quartier Sorge-Bâtiment Génopode, 1015 Lausanne, Switzerland; 5 Chemistry Research Laboratory, University of Oxford, Oxford, OX1 3TA UK; 6 Leicester Institute of Structural and Chemical Biology and Department of Chemistry, University of Leicester, Leicester, LE1 7RH UK

## Abstract

Horizontal gene transfer (HGT) mediated by the spread of plasmids fuels evolution in prokaryotes. Although plasmids provide bacteria with new adaptive genes, they also produce physiological alterations that often translate into a reduction in bacterial fitness. The fitness costs associated with plasmids represent an important limit to plasmid maintenance in bacterial communities, but their molecular origins remain largely unknown. In this work, we combine phenomics, transcriptomics and metabolomics to study the fitness effects produced by a collection of diverse plasmids in the opportunistic pathogen *Pseudomonas aeruginosa* PAO1. Using this approach, we scan the physiological changes imposed by plasmids and test the generality of some main mechanisms that have been proposed to explain the cost of HGT, including increased biosynthetic burden, reduced translational efficiency, and impaired chromosomal replication. Our results suggest that the fitness effects of plasmids have a complex origin, since none of these mechanisms could individually provide a general explanation for the cost of plasmid carriage. Interestingly, our results also showed that plasmids alter the expression of a common set of metabolic genes in PAO1, and produce convergent changes in host cell metabolism. These surprising results suggest that there is a common metabolic response to plasmids in *P. aeruginosa* PAO1.

## Introduction

The movement of genetic information across different bacterial clones is known as horizontal gene transfer (HGT). HGT acts as a major evolutionary force by enabling bacteria to acquire new genes [1, 2]. Plasmids play a crucial role in HGT because they can actively transfer between bacteria through conjugation, spreading accessory genes [3–5]. The most recent and concerning example of how plasmids contribute to bacterial adaptation is the global dissemination of plasmid-mediated antibiotic resistance among clinical pathogens, which represents a main public health emergency worldwide [6, 7]. Despite the adaptive advantages that plasmids may confer to a recipient bacterium, they also produce a fitness cost in their host, generating selection against plasmid-carrying strains [8–10]. Although this cost can be ameliorated through compensatory mutations, it represents one of the main limits for the establishment of plasmids in a new bacterial population [11–17]. Therefore, understanding the origins of these costs is key to predicting and, eventually, preventing the evolution of plasmid-mediated antibiotic resistance.

There are multiple potential sources of costs related to HGT, such as the sequestration of bacterial replication or expression machinery, the biosynthetic cost associated with the new plasmid DNA and proteins, and deleterious interactions between the newly acquired genes and bacterial regulatory networks [18–26]. Although it is clear in many cases that these factors will necessarily translate into an energetic cost for the cell, it is unclear to what extent these translate into fitness costs. One of the most advocated theories is that a central source of fitness cost associated with HGT comes from the translation of proteins from newly acquired genes [8, 27, 28]. This cost is thought to originate from the imbalance between codon usage by the foreign genes and the available tRNA pool in the recipient bacterium [22], leading to reduced translation efficiency in the cell [29–32]. Concurring with this idea, computational studies have shown that codon usage compatibility between newly acquired DNA and the host chromosome favours HGT [27, 28]. However, out of the few experimental studies available on the molecular basis of fitness effects of plasmids [11, 14, 17, 33, 34], only one reported that translation inefficiency is responsible for the cost of HGT [11]. Instead, most experimental studies have found that plasmids impose a fitness cost by interfering with chromosomal replication, leading to the induction of the SOS response [17, 33–36].

In summary, there is a big gap in our understanding of the origin of the costs of HGT in general and of plasmids in particular. Here we used a novel integrative approach to investigate the effects of a diverse collection of plasmids in the opportunistic pathogen *Pseudomonas aeruginosa* PAO1. The goal of this comparative approach was to test for mechanisms that can explain variation in the fitness effects of these plasmids. Specifically, our aims were: (i) to test some of the main mechanisms that have been proposed to explain the cost of plasmid acquisition (translation inefficiency, impaired chromosomal replication and protein biosynthetic costs) and, (ii) to gain a general understanding on the effects of plasmids in the physiology of *P. aeruginosa* PAO1. Our results show that plasmids produce a wide range of fitness effects in PAO1. Although none of the suspected sources of cost could individually explain the fitness effects of the different plasmids, our transcriptomic and metabolomic results showed that plasmids tend to alter preferentially the expression of metabolic genes in PAO1, producing significant and common alterations in the metabolic profiles of the bacterium.

## Material and methods

An extended version of the Material and methods section, including a comprehensive description of every method used in this work, as well as a detailed explanation of the transcriptomic and metabolomic techniques and all the computational analyses, is provided in the Supplementary Information.

### Bacterial strains, plasmids and culture conditions

The plasmids used in this study are described in Table 1. *P. aeruginosa* PAO1 was used as recipient strain. PAO1 WTp*lex*:lux [37] and PAO1 P*lasB*::lux [38] were also used as recipient strains to investigate the effects of plasmids on the SOS response and quorum sensing (QS) system, respectively. Bacterial strains were cultured in LB broth at 37 °C with continuous shaking (225 rpm) and on LB agar plates at 37 °C (Fisher Scientific, NJ, USA). Strains were transformed by electroporation with the different plasmids as previously described [39], using a Gene Pulser apparatus (Bio-Rad). Transformants were selected on LB agar plates containing antibiotics as previously described [15, 16].

**Table 1:** Plasmids used in this study

Name	Group	Size (bp)	Transmission^a^	Origin	Year^b^	Reference
pBS228	IncP-1α	89,147	Mobilizable	Waste water	1981	Haines et al. [55]
Rms149	IncP-6	57,121	Mobilizable	Clinical	1975	Haines et al. [56]
pAKD1	IncP-1β	58,246	Conjugative	Soil	1998	Sen et al. [57]
pAMBL1	RepA/C	26,440	Mobilizable	Clinical	2006	San Millan et al. [58]
pAMBL2	Rep_3	24,133	Non-transmissible	Clinical	2007	San Millan et al. [58]
pNUK73	NA^c^	5128	Non-transmissible	Soil	2003	Itoh et al. [59]

^a^Plasmid classification according to conjugative ability: Conjugative: self-transmissible by conjugation. Mobilizable: able to conjugate using the conjugative machinery of a helper conjugative element. Non-transferable: not able to conjugate or to be mobilized

^b^Year of description

^c^Not applicable. The small plasmid pNUK73 does not belong to a specific plasmid group

### Competitive fitness assays

The fitness of each plasmid-carrying PAO1 clone was determined relative to a PAO1-GFP plasmid-free control strain. The GFP label did not produce a significant reduction in fitness in PAO1 [16]. Competition experiments were performed as previously described [16]. The fitness of the strain carrying the plasmid relative to the PAO1-GFP strain was determined using the formula [40]:

$$W_{{\rm p+}} = {\rm ln}\left(N_{\rm {final,p +}}/N_{\rm {initial,p+}}\right)/{\rm ln}\left(N_{\rm {final,p-}}/N_{\rm {initial,p-}}\right)$$

where *W*_p+_ is the relative fitness of the plasmid-bearing clone, *N*_initial,p+_ and *N*_final,p+_ are the numbers of cells of the plasmid-carrying clone before and after the competition, and *N*_initial,p−_ and *N*_final,p−_ are the numbers of PAO1-GFP cells before and after the competition. As a control group, PAO1 and PAO1-GFP were competed in every experiment. We performed six biological replicates for each competition.

### Statistical analyses

All statistical analyses and production of graphics were performed using R (R Core Team, 2014).

### Data availability

The reads generated in this study have been deposited in the European Nucleotide Archive database with the accession number PRJEB24427 (http://www.ebi.ac.uk/ena/data/view/PRJEB24427).

## Results

### Fitness effects of plasmids in different environments

**Fig. 1:**
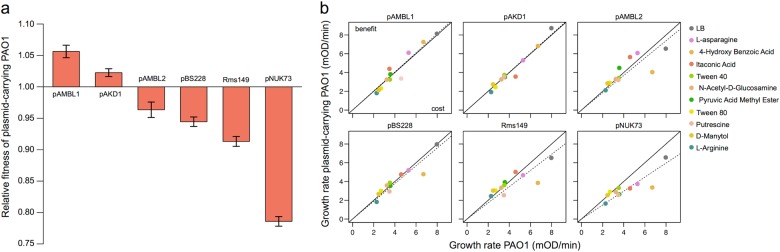
Effects of different plasmids on the fitness of *P. aeruginosa* PAO1. Plasmids produce different fitness effects in PAO1, and these effects tend to remain constant across nutrient environments. **a** Relative fitnesses of plasmid-bearing PAO1 compared to plasmid-free parental strain in LB. Error bars represent the standard error of the mean (SEM, *n* = 5). **b** Comparison of the growth rates of plasmid-free and plasmid-carrying PAO1 in different carbon sources provided by Biolog EcoPlates and in LB broth. The results are the average of six biological replicates of PAO1 and three biological replicates of each of the six plasmid-carrying PAO1 clones. Each panel shows the results for a different plasmid. The colour points indicate growth rates comparisons in the different carbon sources (see legend). The points above the black diagonal line indicate environments where the plasmid produces a benefit, while the points below the line indicate environments where plasmids produce a cost. The dashed line represents the linear regression model of the growth rates of the plasmid-bearing clone against the growth rates of PAO1 over all the environments

We measured the fitness effects of a collection of six antibiotic resistance plasmids with different replication types, from different origins (clinical and environmental) and with different sizes, in *P. aeruginosa* PAO1 (Table 1). First, we performed competition experiments in LB broth to determine the relative fitness of each of the plasmid-carrying strains compared to plasmid-free PAO1 (Fig. 1a). The plasmids produced a variety of fitness effects, from a significant advantage to different degrees of cost. These results matched those of previous studies investigating the fitness effect of these plasmids in PAO1 [15, 16].

One common unanswered question in the field is how much the environment impacts the fitness effects of plasmids (genotype-by-environment interactions). To study the fitness effects of the plasmids in a range of different environments, we used Biolog EcoPlates, which provide 31 different carbon substrates. We observed measurable growth in 10 of the carbon sources, and we determined the growth rate of PAO1 and each plasmid-carrying strain in these environments as well as in LB. In Fig. 1b we compare the growth rates of plasmid-free and plasmid-carrying strains in the different environments. To obtain a general idea of the fitness effects of the plasmids across environments, we regressed the growth rate of the plasmid-bearing clone against the growth rate of PAO1 in the different environments. The slope of this regression measures the deviation in fitness caused by plasmid acquisition and, as such, this metric provides a measure of the fitness effect of the plasmid across environments. Interestingly, the slope of this regression correlated very strongly with competitive fitness in LB, implying that plasmids tend to entail similar costs across different nutrient environments (Pearson’s test, *r* = 0.982, *P* < 0.001, *t* = 10.36, df = 4). Moreover, we observed a positive correlation between the initial growth rate of the plasmid-free strain and the reduction in growth rate imposed by the plasmids across environments (Pearson’s test, *r* = 0.296, *P* = 0.016, *t* = 2.47, df = 64), suggesting that plasmids produced a larger cost under conditions promoting fast bacterial growth.

### Plasmid genes are highly expressed

**Fig. 2:**
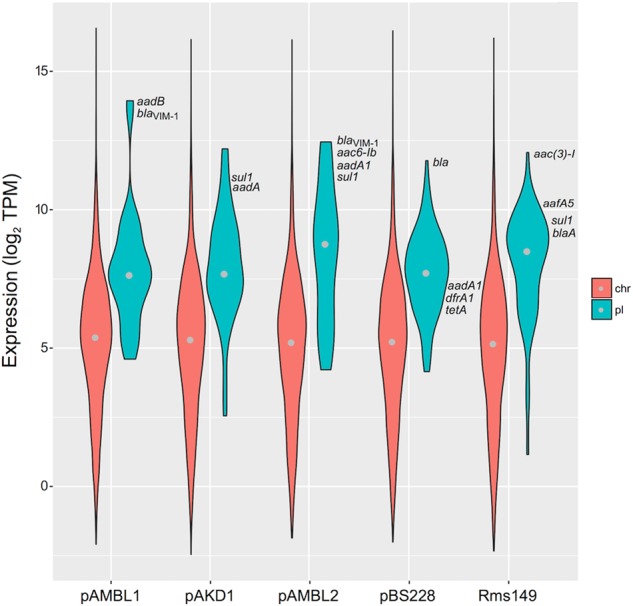
Plasmid genes are highly expressed. Plasmid-encoded genes showed a higher level of expression than chromosome-encoded genes. Violin plot representing the distribution of transcript abundances (in log_2_ of transcripts per million, TPM) of chromosome (red) and plasmid (blue) genes in each plasmid-carrying PAO1. The grey dot represents the median values of the distributions. For clarity, we removed non-expressed chromosomal and plasmid genes in this figure (see main text for statistical analyses including and excluding non-expressed genes and correcting for plasmid copy numbers). Plasmid-encoded antibiotic resistance genes are depicted in the figure according to their level of expression. Note that antibiotic resistance genes are, in general, among the plasmid genes with highest level of expression

To better understand the origin of the fitness effects of plasmids, we performed transcriptomic analyses of five of the plasmid-carrying (pAMBL1, pAMBL2, pAKD1, Rms149 and pBS228) and the plasmid-free PAO1 using RNA-Seq (Supplementary Tables S1 and S2); we have previously used this approach to successfully characterize the origin of the cost produced by the remaining plasmid, pNUK73 [34]. We analysed the transcription profiles of the five plasmids in PAO1 (see Methods, Fig. 2, Supplementary Table S1). In four cases, plasmid genes showed higher levels of expression than the chromosomal genes of their hosts (correcting for plasmid copy number [15], Kolmogorov–Smirnov test, two sided, *P* < 0.0005). The only plasmid that did not show increased gene expression was pAKD1, which is the only conjugative plasmid in the collection. Conjugative plasmids usually control the expression of the conjugative machinery tightly, presumably because expression of conjugative genes carries a large cost [41]. pAKD1 carries more than 20 genes involved in conjugation, which were tightly repressed (Supplementary Table S1). To compare the expression of genes with detectable levels of transcription in pAKD1 and PAO1 chromosome, we removed non-expressed chromosomal and plasmid genes from the analysis. After this correction, pAKD1 genes also showed a higher level of expression than those from the chromosome (Kolmogorov–Smirnov test, two sided, *P* < 0.0005).

Interestingly, antibiotic resistance genes were among the genes with highest level of expression in the plasmids (Fig. 2, Supplementary Table S1). Most of the plasmid-carried antibiotic resistance genes were located in integrons, which are genetic platforms able to capture promoterless genes called cassettes and express them in a decreasing gradient from a single strong promoter [42]. Integrons are highly prevalent on plasmids (mobile integrons), and they are usually associated with antibiotic resistance genes [43]. Our results showed that integrons drive high-level expression of plasmid resistance genes. Despite the high expression level of plasmid genes, the RNA reads mapping to plasmids only represented between 1.75 and 2.85% of the total number of reads in the cell (Table S3), and we did not find a negative correlation between the amount of reads mapping to plasmids and the relative fitness of the plasmid-carrying PAO1 (Pearson’s test, *r* = 0.408, *P* = 0.495, *t* = 0.775, df = 3). This result suggests that the energetic cost associated with RNA synthesis alone cannot explain the cost imposed by plasmids.

### Plasmids alter the expression of metabolic genes in PAO1

**Fig. 3:**
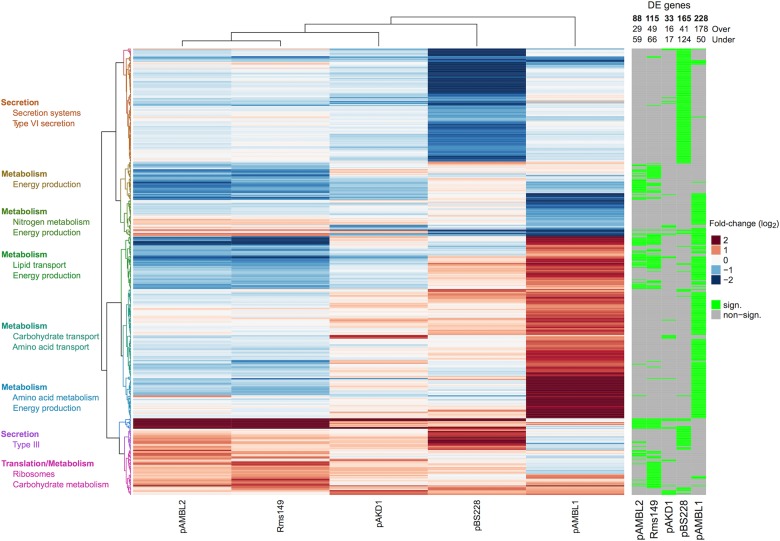
Differentially expressed genes in the chromosome of plasmid-carrying PAO1. Differential expression of chromosomal genes in PAO1 carrying different plasmids, compared to plasmid-free PAO1. In this heatmap we present the genes that are significantly differentially expressed (DE) in at least one of the plasmid-carrying clones compared to plasmid-free PAO1. The genes significantly DE are indicated by the green bars to the right of the figure. Red bars in the heatmap indicate those genes that are over-expressed and blue bars those under-expressed. The intensity of the colour is proportional to the level of differential expression, as indicated in the colour legend (log_2_ fold-change). Genes upregulated or downregulated by more than four fold are coloured at the same (maximum) intensity. To the left of the figure we indicated functions significantly enriched in the different clusters of genes formed in the heatmap. Note that “metabolism” is the function most commonly enriched, indicating that plasmids tend to alter the expression of metabolic genes in PAO1

We compared the expression of chromosomal genes in the different plasmid-carrying strains to that of plasmid-free PAO1. Plasmids altered the expression (over-expression or under-expression) of chromosomal genes [hereafter, differentially expressed (DE) genes]; from 34 genes in PAO1/pAKD1 to 228 in PAO1/pAMBL1 (Fig. 3, Supplementary Table S2, Supplementary Figure S2). Interestingly, we observed that even though plasmids produced very different effects on the transcription profile of PAO1, there was a significant subset of genes that were DE in common in the different plasmid-carrying PAO1 (Supplementary Figure S3). For example, there were 38 DE genes in common in at least three plasmid-carrying strains (Supplementary Table S4), while chance alone would predict less than one (Fisher’s test, *P* < 0.0001, df = 1). Notably, when we compared these results with our previous work involving pNUK73, we found that 29 out of those 38 genes were also DE in PAO1/pNUK73 (Supplementary Table S4) [34].

To better understand the effects of plasmids on PAO1, we performed a functional enrichment analysis of the DE genes in the chromosomes of the plasmid-carrying clones (Fig. 3, Supplementary Table S5). PAO1/pAKD1 did not show an enrichment of genes DE in any particular category. pAMBL1 and pAMBL2 preferentially altered the expression of metabolic genes in the chromosomes of their hosts. Rms149 altered the expression of metabolic genes and genes related to ribosomes and translation. Finally, DE genes in PAO1/pBS2388 were enriched in protein secretion systems, which are key virulence factors in *P. aeruginosa*. When we analysed all the DE genes in all the strains combined, there was again an overrepresentation of genes involved in metabolism. Moreover, the 38 DE genes in common in at least three plasmid-carrying strains were also enriched in metabolic functions (Supplementary Table S5). Together, these results suggest two conclusions: (i) plasmids tend to alter the expression of metabolic genes in the chromosome of PAO1 and, (ii) there is a group of genes, enriched in metabolic functions, which are DE in response to the presence of different plasmids.

To dissect the results obtained in the functional enrichment analysis, we scanned the profiles of expression of the metabolic genes that were DE in the plasmid-carrying strains [44]. This analysis revealed interesting patterns: all plasmids, apart from pAMBL1, which produces the largest fitness benefit, were associated with an over-expression of genes involved in glutamine synthesis (PA5506–PA5509) [45]. Conversely, in PAO1/pAMBL1 there was a group of over-expressed metabolic genes, which were either non-affected or under-expressed in the presence of the remaining plasmids. These genes were responsible for valine, leucine and isoleucine degradation (PA2012–PA2014), tyrosine and phenylalanine metabolism (PA2007–PA2009), glycine, serine and threonine metabolism (PA2442–PA2446), benzoate degradation (PA1999–PA2000) and fatty acid metabolism (PA2001 and PA0744). Interestingly, PA4918, which encodes a putative nicotinamidase [46], was the only gene whose differential expression (over-expression) was specifically associated with the presence of costly plasmids.

### Analysing potential sources of plasmid cost

#### Impaired chromosomal replication is not a general effect of plasmids in PAO1

**Fig. 4:**
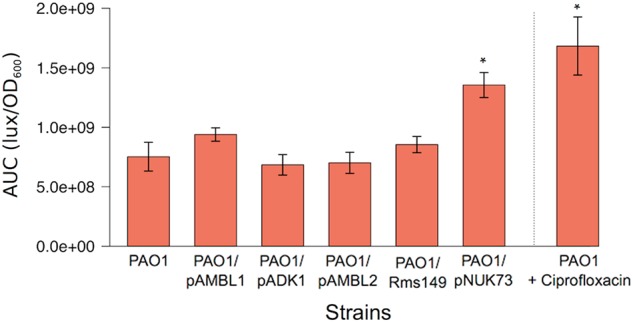
Plasmids do not mediate activation of SOS response. Not all plasmids induce the SOS response in PAO1. The figure represents the area under the curve of luminescence production over OD_600_ [AUC (lux/OD_600_)] during the exponential phase of the growth curves of PAO1 WTp*lex*:lux reporter strain, which encodes the luciferase operon under the control of an SOS inducible promoter (PAO1 in the figure). We also present the AUC (lux/OD_600_) of the different plasmid-carrying WTp*lex*:lux, and a control with the plasmid-free WTp*lex*:lux growing in the presence of a sub-inhibitory concentration of the SOS-inducing antibiotic ciprofloxacin (45 μg/L). The bars indicate the average of eight biological replicates and the error bars indicate the standard error. Asterisks indicate significant differences in SOS activation (Tukey’s post-hoc test, *P* < 0.05). As expected, plasmid pNUK73 and the presence of ciprofloxacin induced the SOS response. However, none of the remaining plasmids produced the activation of the stress response

Previous experimental studies have shown that plasmids can interfere with chromosomal replication, which may entail a large fitness cost [17, 34–36]. One symptom of stalled chromosomal replication is increased expression of the SOS pathway in response to single-stranded DNA. To test for plasmid-mediated inhibition of chromosomal replication, we looked for an increase in the expression of SOS pathway genes. Our transcriptomic data did not show an overexpression of SOS-mediated genes in any of the five plasmid-carrying bacteria (Supplementary Table S2). To confirm these results we introduced the plasmids, including pNUK73 (which activates the SOS response [34]), into a PAO1 reporter strain carrying a luciferase operon under the control of an SOS inducible promoter [37]. We could not introduce plasmid pBS228 into the reporter strain, because this plasmid has very poor electroporation efficiency due to its large size. The production of luminescence was measured during the exponential phase of the growth curves of the different strains. We included a positive control for which we added sub-inhibitory concentrations of the SOS-inducing antibiotic ciprofloxacin to the plasmid-free reporter strain. Our results revealed significant differences in SOS activation among strains (Fig. 4, analysis of variance: *P* *<* 0.0001, *F* = 74.73, df = 6, 49). As expected, ciprofloxacin and pNUK73 produced an increase in luminescence production compared to the plasmid-free PAO1 (Tukey’s post-hoc test, *P* *<* 0.0001). The remaining plasmids, however, produced no significant changes (Tukey’s post-hoc test, *P* > 0.05). These results suggest that impaired chromosomal replication is not a general effect of plasmids in PAO1.

#### Translation inefficiency as a source of plasmid cost

Codon usage imbalance between horizontally acquired genes and the host chromosomal genes can lead to inefficient ribosome allocation and ribosome pausing [22]. To investigate if translation inefficiency was responsible for the fitness costs associated with the plasmids under study, we scanned the expression profiles of chromosomal genes of plasmid-carrying strains and looked for signatures of increased translational demand. Only PAO1/Rms149 showed an overexpression of genes involved in translation and ribosomes compared to plasmid-free PAO1 (Fig. 3, Supplementary Table S5), which could be the consequence of an increase in translational demand [11]. Interestingly, Rms149 produced the highest cost out of the five plasmids analysed by RNA-Seq in this study. However, the remaining plasmids showed no significant effect on the expression levels of translation-associated genes.

To obtain an estimation of the codon usage compatibility between plasmids and PAO1 genes, we calculated the distribution of codon adaptation index (CAI) for plasmid genes (Supplementary Figure S4). The CAI quantifies the similarity between the synonymous codon usage of a given gene and that of a subset of highly expressed genes in *P. aeruginosa* PAO1 (see Methods). CAI therefore provides a proxy for the translational burden imposed by the gene; genes with lower CAI carry rare codons that may stall ribosomes. However, it is important to take into account the expression levels of plasmid genes to obtain a realistic idea of the translational burden imposed by plasmids. Therefore, we weighted the CAI value of each plasmid gene by its expression level. We did not find a positive correlation between the median corrected CAI values of plasmid genes and the relative fitness of plasmid-carrying strains (Pearson’s test, *r* = −0.150, *P* = 0.809, *t* = −0.262, df = 3, see Supplementary Tables S6 and S7 for an analysis of CAI and expression levels of plasmid genes). Taken together, these results indicate that translation inefficiency produced by plasmid transcripts is probably not a general cause of fitness cost in *P. aeruginosa* PAO1.

#### Biosynthetic costs of plasmid-encoded proteins

Most of the bioenergetic cost associated with expressing genes comes from protein synthesis [20, 47]. Therefore, the cost associated with synthesizing newly acquired proteins may be an important limit to HGT. To determine the burden associated with synthesizing plasmid-encoded proteins, we calculated the biosynthetic cost of both plasmid and chromosomal proteins for each plasmid-carrying strain (correcting for the gene expression levels, see Methods and Supplementary Table S8). Our results showed that the biosynthetic cost of plasmid-encoded proteins ranged from 2.46 to 3.72% of the total costs associated with protein synthesis of the cells (Supplementary Table S9). However, the relative fitness of the plasmid-carrying clones did not negatively correlate with the relative cost of plasmid-encoded proteins (Pearson’s test, *r* = 0.225, *P* = 0.716, *t* = 0.40, df = 3) nor with the biosynthetic cost associated with plasmid-induced change in chromosomal gene expression (Pearson’s test, *r* = 0.858, *P* = 0.063, *t* = 2.89, df = 3).

### Metabolic analysis of plasmid-carrying PAO1

**Fig. 5:**
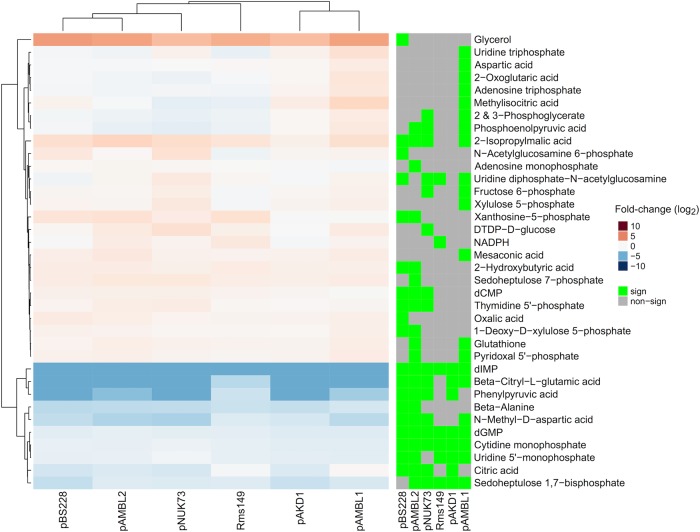
Change in abundance of identified metabolites. Different plasmids produce similar changes in the abundance of the identified metabolites in the host bacterium *P. aeruginosa* PAO1. Heatmap representing those identified metabolites with significant differences in abundance in at least one of the plasmid-carrying PAO1 compared to plasmid-free PAO1 (indicated by the green squares to the right of the figure). Metabolites with higher abundance are represented in red, and metabolites with lower abundance are represented in blue. The intensity of the colour is proportional to the differences in metabolite concentration, as indicated in the colour legend (log_2_ fold-change). We performed five replicates per strain for the metabolomic analysis

Transcriptomic data revealed that plasmids preferentially altered the expression of metabolic genes in PAO1. We applied mass spectrometry to investigate how these alterations impacted the metabolic profiles of plasmid-carrying clones. We performed metabolic profiling of parental PAO1 and all six plasmid-carrying PAO1, including PAO1/pNUK73 (since we did not investigate the metabolic profile of this strain in our previous work [34]). We detected more than 5000 compounds across the samples and were able to identify 97 based on matching criteria with authentic standards. To understand the metabolic effects caused by the plasmids, we compared the abundance of each compound in the plasmid-carrying strains with its abundance in the plasmid-free PAO1 strains (Supplementary Table 10). The different plasmids impacted the concentrations of multiple compounds and, interestingly, the changes in the metabolic profiles produced by the different plasmids showed a high degree of parallelism, both in the compounds affected and in the direction of the changes (Supplementary Figure S5 and Fig. 5). The different plasmids affected the abundance of a common subset of compounds. For example, out of the total compounds detected, 462 showed a significant change in abundance in common in at least four of the six plasmid-carrying clones, when chance alone predicted approximately nine (Chi-squared test, *P* < 0.0001, *χ*^2^ = 452.43, df = 1, Supplementary Figure S5, Supplementary Table S11). For the identified metabolites, 11 were significantly altered in at least four plasmid-carrying clones in common, while chance predicted less than one (Fisher’s test, *P* = 0.005, df = 1, Fig. 5, Supplementary Table S11).

Although analysis of the metabolomics data is complicated by the low number of identified metabolites, it is possible to tentatively recognise some general trends. Firstly, it is notable that many of the identified nucleotides have altered abundance in the plasmid-carrying clones relative to PAO1. While this may suggest dysregulated nucleotide biosynthesis and/or metabolism in the clones, it is also likely that expression of plasmid genes increases the demand for nucleotides, potentially leading to a re-buffering of nucleotide concentrations. It is interesting that the RNA nucleotides cytidine monophosphate and uridine monophosphate appear down-regulated in the plasmid-carrying clones, whereas the equivalent deoxynucleotides are either up-regulated or unchanged. This observation may imply an increase in RNA biosynthesis in the plasmid-carrying clones. While plasmid-induced protein biosynthesis does not seem to cause a significant fitness cost (see above), it is possible it induces toxic knock-on effects. For example, the PAO1/pAMBL1 clone may overcome the cost of increased protein biosynthesis by over-expressing genes involved in amino acid metabolism (as we observed), thereby removing potentially toxic levels of amino acids released after protein degradation. It should also be noted that levels of pyrimidine nucleotides may be affected by the common over-expression of glutamine biosynthesis genes (see above); glutamine is a co-substrate of cytidine synthetase, which interconverts uridine and cytidine triphosphates.

## Discussion

In this work we applied, for the first time, an integrative approach combining phenotypic characterization with transcriptomic and metabolomic analyses to decipher the origin of the fitness effects produced by plasmids. Our results suggest that the fitness effects produced by plasmids are multifactorial, as none of the different causes analysed could explain the fitness effects individually. However, several lines of evidence indicate that, as expected, undomesticated plasmids produce a larger cost in *P. aeruginosa* PAO1. For example, using GC content as a simple proxy for phylogenetic proximity, we found a clear correlation between plasmid–chromosome differences in GC content and the cost imposed by the plasmid (Supplementary Figure S6, Pearson’s test, *r* = 0.969, *P* *=* 0.001, *t* = 7.87, df = 4). Moreover, the plasmid producing the least fitness alteration in PAO1, pAKD1, was also the plasmid producing the least alteration at molecular and physiological levels (most “domesticated”): namely low expression levels of plasmid genes, low protein biosynthetic cost, small alteration of transcription and metabolic profiles of the host, and a GC and codon usage similar to those in PAO1. These results are in line with previous research showing that high-level expression or codon usage incompatibility hampers HGT [25, 27, 28, 48], while mechanisms controlling expression of plasmid genes, such as H-NS proteins, facilitate HGT [49–51]. Broad host range IncP-1 plasmids, such as pAKD1, possess control circuits that minimize the expression of genes coding for propagation functions after plasmid establishment [52], and they usually produce low fitness costs across different bacterial hosts [53].

The most novel finding of this study is that different plasmids alter the expression of a common set of metabolic genes in PAO1, and lead to convergent changes in host cell metabolism (Figs. 3 and 5 and Supplementary Figure 5). Transcriptomic results revealed interesting differences between the expression profiles of metabolic genes of plasmid-carrying strains. All plasmids, except pAMBL1 (which produces the greatest fitness advantage), were associated with an over-expression of genes involved in glutamine synthesis. Notably, glutamine is thought to play a key role in the nitrogen control of *P. aeruginosa* [54]. On the other hand, the beneficial pAMBL1 showed a unique transcription profile, with over-expression of genes involved amino acid and fatty acid metabolism. A clear correlation is also observed between fitness cost and expression of an as yet uncharacterised nicotinamidase, implying its enzyme activity (i.e. conversion of nicotinamide to niacin and ammonia) could induce a fitness cost. It is tempting to speculate on how these changes in expression of chromosomal genes may be responsible for the fitness effects of plasmids, however, we prefer to remain cautious in our interpretations, because although these changes could be the cause of altered fitness, they could also be the consequence of those fitness effects. Future work will be needed to investigate these possibilities.

Our transcriptomic data are supported by metabolomic analyses on the PAO1 clones, which reveal striking changes in metabolite concentrations, particularly in the levels of nucleotides. While we are cautious not to over-interpret our results, they tantalizingly suggest that there may be a common metabolic response to plasmids in *P. aeruginosa* PAO1. It is important to highlight that this metabolic response cannot explain variation in fitness across the plasmid-carrying PAO1, because it is produced by both costly and beneficial plasmids. Therefore, the link between these common metabolic effects and fitness is not completely clear. One possible explanation is that bacteria respond to the presence of plasmids by altering metabolism to compensate for the new physiological requirements. In some cases, such as pAKD1 and pAMBL1, these alterations could be sufficient to eliminate the burden of plasmid carriage, and there is no net cost associated with plasmid acquisition. In other cases, such as pBS228 and Rms149, these metabolic changes could be insufficient to compensate for the cost of plasmid carriage, and plasmid carriage reduces fitness. It is not obvious which genes are responsible for regulating this response, as either plasmid genes or chromosomal genes could theoretically control it. However, given the diversity of plasmids employed in this study, the most parsimonious explanation would be that chromosomal genes regulate this response.

One limitation of this work is the fact that although the plasmid collection used here covers a wide range of plasmid families and sizes, we did not include a megaplasmid (>100 kb), which are relatively common in *P. aeruginosa*. Another limitation is that in this work we used a QS-deficient PAO1 strain as model system (see Methods). However, we argue that since the plasmids in our collection do not disturb QS (see control experiment in Supplementary Figure 7), this fact should not affect the interpretation of our results.

In conclusion, our results reveal new insights on the effects of plasmids in bacteria and on the routes towards plasmid domestication. Crucially, this work paves the way for new research on the molecular and evolutionary consequences of plasmid carriage, which may help to discover new targets in the fight against the dissemination of plasmid-mediated antibiotic resistance.

## Electronic supplementary material

Supplementary Table S1

Supplementary Table S2

Supplementary Table S3.

Supplementary Table S4

Supplementary Table S5

Supplementary Table S6

Supplementary Table S7

Supplementary Table S8

Supplementary Table S9

Supplementary Table S10

Supplementary Table S11

Supplementary Table S11

Supplementary Information Figures

Supplementary Information
