# Supplementary material for: Integrative analysis of fitness and metabolic effects of plasmids in Pseudomonas aeruginosa PAO1
Source: ISME J. 2018 Aug 10;12(12):3014–24. doi: 10.1038/s41396-018-0224-8 (PMC6246594; doi:10.1038/s41396-018-0224-8)
Supplement: Supplementary file 3 — Supplementary Table S3. [file 41396_2018_224_MOESM3_ESM.docx]

**Supplementary Table S3.** Proportion of reads mapping to plasmids out of the total read counts in the cell.

| Plasmid | Reads from plasmid (%) | Relative fitness of plasmid-carrying PAO1 |
| --- | --- | --- |
| pAMBL1 | 2.846 | 1.056 |
| pAKD1 | 1.892 | 1.022 |
| pAMBL2 | 1.745 | 0.963 |
| pBS228 | 2.623 | 0.944 |
| Rms149 | 1.927 | 0.913 |
