# Supplementary material for: Integrative analysis of fitness and metabolic effects of plasmids in Pseudomonas aeruginosa PAO1
Source: ISME J. 2018 Aug 10;12(12):3014–24. doi: 10.1038/s41396-018-0224-8 (PMC6246594; doi:10.1038/s41396-018-0224-8)
Supplement: Supplementary file 7 — Supplementary Table S7 [file 41396_2018_224_MOESM7_ESM.docx]

**Supplementary Table S7.** Expression of plasmid genes with different codon usages.

| CAI | pAKD1 | pAMBL1 | pAMBL2 | pBS228 | Rms149 |
| --- | --- | --- | --- | --- | --- |
| low | 7.77% | 79.40% | 69.50% | 32.51% | 20.48% |
| medium | 60.37% | 20.60% | 28.20% | 56.27% | 79.16% |
| high | 31.86% | 0 | 2.31% | 11.22% | 0.37% |

Percentage of TPM for each plasmid that fall inside each CAI category. CAI categories were done using the mean CAI for plasmid genes (0.498) and adding or subtracting 1 standard deviation (0.110). For each CAI category, and each plasmid, we summed the TPM of all the genes that fall in that specific CAI category, and we calculated the fraction that this represents from the total number of TPM for a given plasmid.
