# Supplementary material for: Integrative analysis of fitness and metabolic effects of plasmids in Pseudomonas aeruginosa PAO1
Source: ISME J. 2018 Aug 10;12(12):3014–24. doi: 10.1038/s41396-018-0224-8 (PMC6246594; doi:10.1038/s41396-018-0224-8)
Supplement: Supplementary file 9 — Supplementary Table S9 [file 41396_2018_224_MOESM9_ESM.docx]

**Supplementary Table S9.** Biosynthetic cost of proteins expressed from plasmids (relative to the total protein biosynthetic cost in the cell, corrected by expression).

| Plasmid | Biosynthetic cost from plasmid (%) | Relative fitness of plasmid-carrying PAO1 |
| --- | --- | --- |
| pAMBL1 | 3.699 | 1.056 |
| pAKD1 | 2.457 | 1.022 |
| pAMBL2 | 3.724 | 0.963 |
| pBS228 | 3.487 | 0.944 |
| RmS149 | 2.512 | 0.913 |
