# Supplementary material for: Integrative analysis of fitness and metabolic effects of plasmids in Pseudomonas aeruginosa PAO1
Source: ISME J. 2018 Aug 10;12(12):3014–24. doi: 10.1038/s41396-018-0224-8 (PMC6246594; doi:10.1038/s41396-018-0224-8)
Supplement: Supplementary file 12 — Supplementary Table S11 [file 41396_2018_224_MOESM12_ESM.docx]

Table 1. Plasmids used in this study

| Name | Group | Size (bp) | Transmission^1^ | Origin | Year^2^ | Reference |
| --- | --- | --- | --- | --- | --- | --- |
| pBS228 | IncP-1α | 89,147 | Mobilizable | Waste water | 1981 | (Haines et al 2007) |
| Rms149 | IncP-6 | 57,121 | Mobilizable | Clinical | 1975 | (Haines et al 2005) |
| pAKD1 | IncP-1β | 58,246 | Conjugative | Soil | 1998 | (Sen et al 2011) |
| pAMBL1 | RepA/C | 26,440 | Mobilizable | Clinical | 2006 | (San Millan et al 2015a) |
| pAMBL2 | Rep_3 | 24,133 | Non-transmissible | Clinical | 2007 | (San Millan et al 2015a) |
| pNUK73 | NA^3^ | 5,128 | Non-transmissible | Soil | 2003 | (Itoh et al 2003) |

^1^ Plasmid classification according to conjugative ability: Conjugative: self-transmissible by conjugation. Mobilizable: able to conjugate using the conjugative machinery of a helper conjugative element. Non-transferable: not able to conjugate or to be mobilized.

^2^ Year of description.

^3^ Not applicable. The small plasmid pNUK73 does not belong to a specific plasmid group.

**Supplementary Tables**

**Supplementary Table S1.** Transcriptional profiles of plasmids.

Levels of expression (in transcripts per million, TPM) of plasmid genes in the different plasmid-carrying PAO1.

**Supplementary Table S2.** Chromosomal genes DE due to presence of plasmids.

Table with the genes DE (under and over) for each of the 5 combinations of PAO1/plasmid compared to plasmid-free PAO1. The cut-off for classifying a gene as DE is padj <0.05.

| Plasmid | Reads from plasmid (%) | Relative fitness of plasmid-carrying PAO1 |
| --- | --- | --- |
| pAMBL1 | 2.846 | 1.056 |
| pAKD1 | 1.892 | 1.022 |
| pAMBL2 | 1.745 | 0.963 |
| pBS228 | 2.623 | 0.944 |
| Rms149 | 1.927 | 0.913 |

**Supplementary Table S3.** Proportion of reads mapping to plasmids out of the total read counts in the cell.

**Supplementary Table S4.** Genes DE in common in plasmid-carrying PAO1.

Group of 38 genes DE in common in at least three of the five plasmid-carrying PAO1 analysed in this study. We also show the results from PAO1/pNUK73 from a previous analysis (San Millan et al 2015). Genes significantly DE are indicated with the value “1”, and those showing no significant DE are indicated with “0”. “+” indicates over-expression and “-“ under-expression.

**Supplementary Table S5**. Functional enrichment analysis for DE genes.

Functional enrichment analysis of those genes DE in each plasmid-carrying PAO1 independently and in combination (all genes DE, under and over). The analysis is also shown for the genes DE in common in at least three plasmid-carrying PAO1 and for the groups of genes from the clusters in Figure 3.

**Supplementary Table S6.** CAI values for plasmids and PAO1 genes.

CAI and expression values for plasmid genes. CAI values for all PAO1 genes.

**Supplementary Table S7.** Expression of plasmid genes with different codon usages.

| CAI | pAKD1 | pAMBL1 | pAMBL2 | pBS228 | Rms149 |
| --- | --- | --- | --- | --- | --- |
| low | 7.77% | 79.40% | 69.50% | 32.51% | 20.48% |
| medium | 60.37% | 20.60% | 28.20% | 56.27% | 79.16% |
| high | 31.86% | 0 | 2.31% | 11.22% | 0.37% |

Percentage of TPM for each plasmid that fall inside each CAI category. CAI categories were done using the mean CAI for plasmid genes (0.498) and adding or subtracting 1 standard deviation (0.110). For each CAI category, and each plasmid, we summed the TPM of all the genes that fall in that specific CAI category, and we calculated the fraction that this represents from the total number of TPM for a given plasmid.

**Supplementary Table S8.** Biosynthetic cost of PAO1 and plasmids proteins.

Biosynthetic cost (measured in ~P, activated phosphate; energy costs were obtained from (Wagner 2005) and (Akashi and Gojobori 2002)) of the proteins encoded in PAO1 chromosome and in the different plasmids of the study. To correct for gene expression levels we weighted the biosynthetic cost by the expression levels (see methods).

**Supplementary Table S9.** Biosynthetic cost of proteins expressed from plasmids (relative to the total protein biosynthetic cost in the cell, corrected by expression).

| Plasmid | Biosynthetic cost from plasmid (%) | Relative fitness of plasmid-carrying PAO1 |
| --- | --- | --- |
| pAMBL1 | 3.699 | 1.056 |
| pAKD1 | 2.457 | 1.022 |
| pAMBL2 | 3.724 | 0.963 |
| pBS228 | 3.487 | 0.944 |
| RmS149 | 2.512 | 0.913 |

**Supplementary Table S10.** Comparison of metabolite abundance between plasmid-carrying PAO1 and plasmid-free PAO1.

Metabolites presenting a significant difference (q-value<0.05) in abundance between the different plasmid-carrying PAO1 and the plasmid-free strain. Both identified and non-identified metabolites are presented.

**Supplementary Table S11.** Common metabolites with different abundance in plasmid-carrying PAO1.

Group of metabolites showing differences in abundance in common in plasmid-carrying PAO1 compared to plasmid-free PAO1. Both non-identified (first tab) and identified metabolites (second tab) are presented. Metabolites with different abundance are indicated with the value “1”, and those showing no significant difference are indicated with “0”.
