# Supplementary material for: Integrative analysis of fitness and metabolic effects of plasmids in Pseudomonas aeruginosa PAO1
Source: ISME J. 2018 Aug 10;12(12):3014–24. doi: 10.1038/s41396-018-0224-8 (PMC6246594; doi:10.1038/s41396-018-0224-8)
Supplement: Supplementary file 13 — Supplementary Information Figures [file 41396_2018_224_MOESM13_ESM.docx]

**Supplementary Figures**

**Supplementary Figure S1.** PCA plot discarding batch effects in RNA-Seq replicates.

PCA plot from the RNA-Seq results using DESeq2 to check for batch effects. The two biological replicates from each strain grouped together, showing minimal batch effect.

**Supplementary Figure S2.** Direction of changes in expression of chromosomal genes of plasmid-carrying PAO1.

Violin plot representing the distribution of the fold changes in expression of chromosomal genes in the different plasmid-carrying PAO1 compared to plasmid-free PAO1. Only significant differentially expressed genes (DE) are represented in the figure (P-adjusted< 0.05). The grey dot represents the median value of the distribution. Costly plasmids (pAMBL2, pBS228 and Rms149) preferentially produced down-regulation of chromosomal genes (values of log2 fold change below 0), whereas beneficial plasmids (pAMBL1 and pAKD1) tended to entail up-regulation.

**Supplementary Figure S3.** Genes differentially expressed (DE) in common in the different plasmid-carrying PAO1.


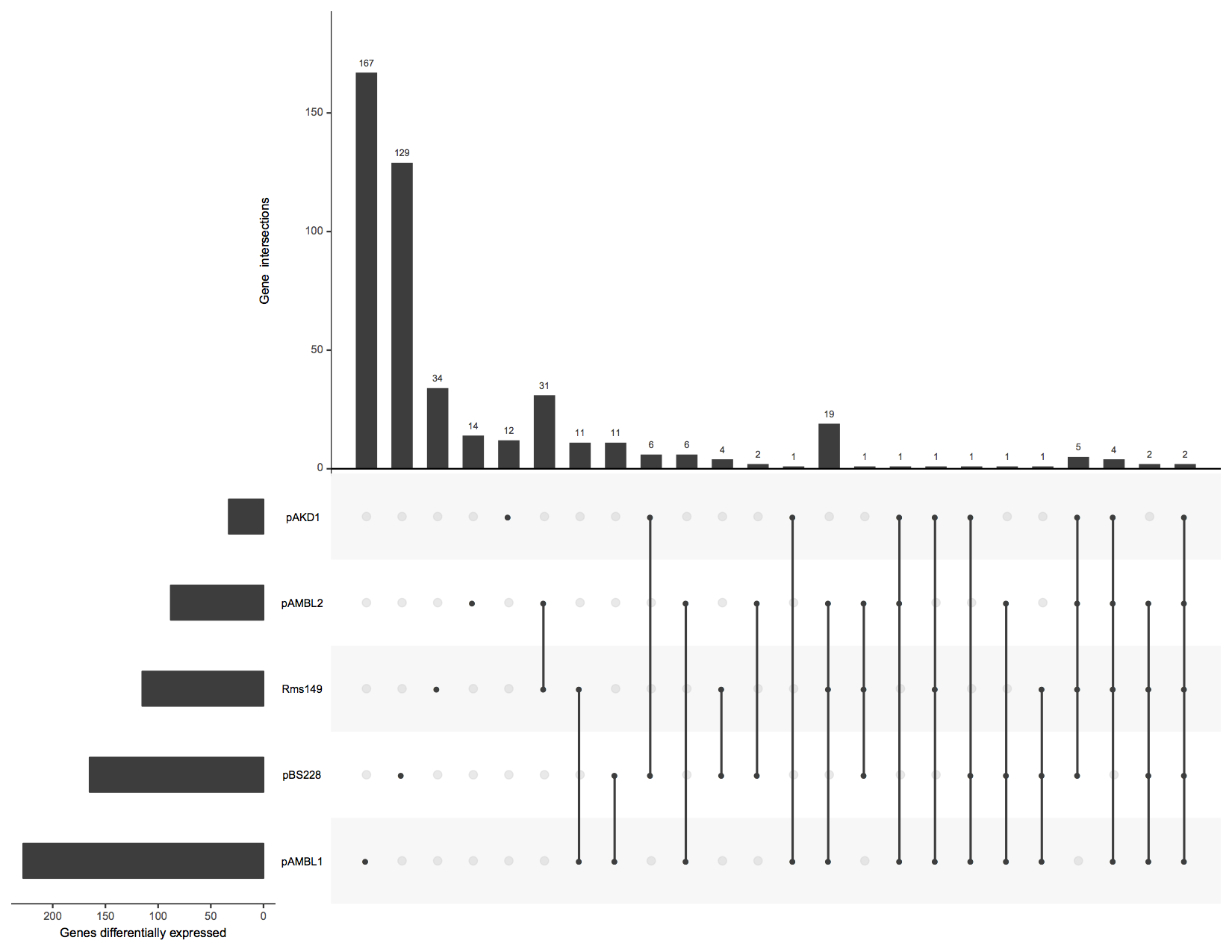


Diagram representing number of DE genes in each PAO1/plasmid combination, and shared DE genes between the different combinations. Note that each DE gene is only included in one category (bar), as in a Venn diagram.

**Supplementary Figure S4.** Distribution of codon adaptation index (CAI) values for chromosomal and plasmid genes.

Violin plot representing the distribution of CAI values for all the genes in PAO1 chromosome (in red) and CAI values for the genes encoded in the different plasmids (see colour legend). The grey dot represents the median value of the distribution.

**Supplementary Figure S5**. Plasmids produce a parallel metabolic response in PAO1.


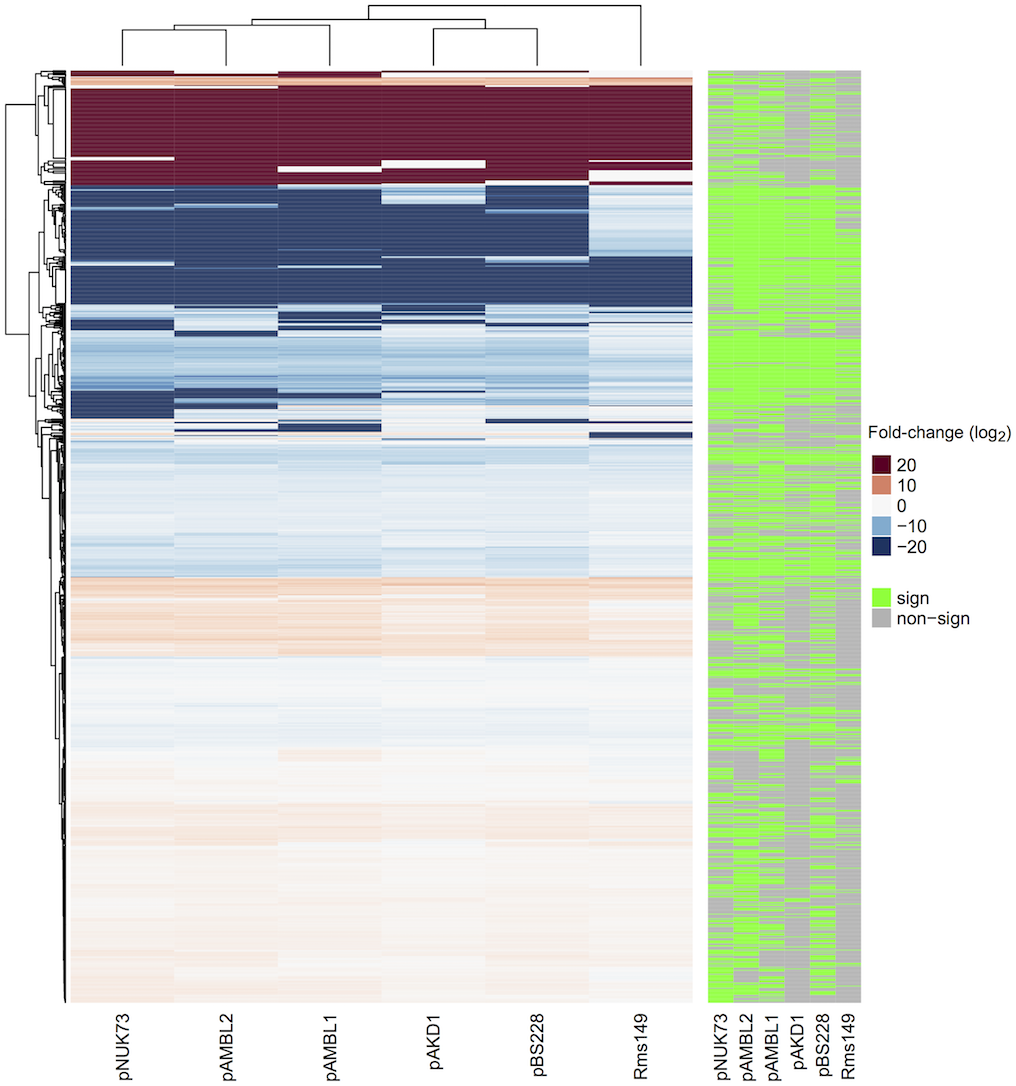


Different plasmids produce similar changes in metabolite abundance in the host bacterium *P. aeruginosa* PAO1. Heatmap representing those compounds with significant differences in abundance in at least one of the plasmid-carrying PAO1 compared to plasmid-free PAO1 (indicated by the green bars to the right of the figure). Compounds with higher abundance are represented in red, and compounds with lower abundance are represented in blue. The intensity of the colour is proportional to the differences in concentration, as indicated in the colour legend (log_2_ fold-change). Compounds with an increase or decrease in abundance higher than 2^20^ fold are coloured at the same (maximum) intensity. We performed 5 replicates per strain for the metabolomic analysis.

**Supplementary Figure S6.** Correlation between plasmid GC content and relative fitness of the plasmid-bearing PAO1.

Plasmids with low GC content produce higher costs than plasmids with high GC content (more similar to the one from the host bacterial strain *P. aeruginosa* PAO1: 66.6%). There is a positive correlation between relative fitness of plasmid-carrying strain and the GC content of the plasmid (Pearson's test, r= 0.969, *P=* 0.001, t= 7.87, df= 4).

**Supplementary Figure S7**. Plasmids do not alter QS system in PAO1.


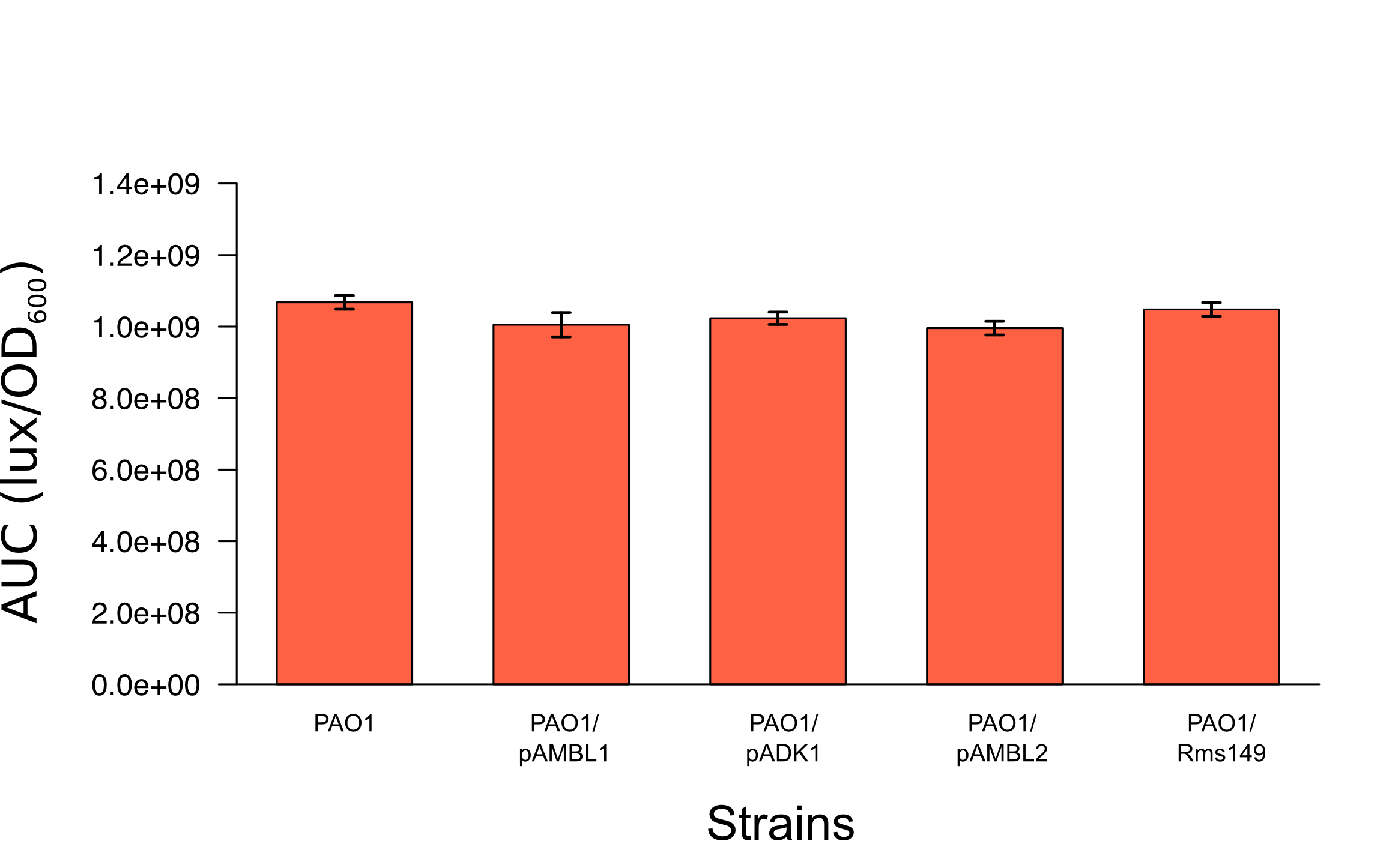


The plasmids in our collection do not affect the QS system in PAO1. The figure represents the area under the curve of luminescence production over OD600 [AUC (lux/OD600)] during the growth curves of PAO1 P*lasB*::lux reporter strain, which encodes a chromosomal *luxCDABE* fusion to the promoter of the *lasB* gene (P*lasB*) (PAO1 in the figure). We also present the AUC (lux/OD600) of the different plasmid-carrying P*lasB*::lux. We used pAKD1, pAMBL1, pAMBL2 and Rms149 plasmids (pBS228 has very poor electroporation efficiency due to its large size and we were not able to obtain a transformed strain). The bars indicate the average of 4 biological replicates (of six technical replicates each) and the error bars indicate the standard deviation.
