# Supplementary material for: Integrative analysis of fitness and metabolic effects of plasmids in Pseudomonas aeruginosa PAO1
Source: ISME J. 2018 Aug 10;12(12):3014–24. doi: 10.1038/s41396-018-0224-8 (PMC6246594; doi:10.1038/s41396-018-0224-8)
Supplement: Supplementary file 14 — Supplementary Information [file 41396_2018_224_MOESM14_ESM.docx]

**Supplementary Information**

**Integrative analysis of fitness and metabolic effects of plasmids in *Pseudomonas aeruginosa* PAO1**

Alvaro San Millan^1,2,Ψ,^*, Macarena Toll-Riera^1,3,4,Ψ,^*, Qin Qi^1^, Alex Betts^1^, Richard J Hopkinson^5,6^, James McCullagh^5^, R Craig MacLean^1^.

^1^ Department of Zoology, University of Oxford, OX2 6GG, Oxford, United Kingdom.

^2^ Department of Microbiology, Hospital Universitario Ramon y Cajal (IRYCIS) and Network Research Centre for Epidemiology and Public Health (CIBERESP), 28034, Madrid, Spain.

^3^ Department of Evolutionary Biology and Environmental Studies, University of Zurich, Zurich CH-8057, Switzerland.

^4^ Swiss Institute of Bioinformatics, Quartier Sorge-Bâtiment Génopode, Lausanne 1015, Switzerland.

^5^ Chemistry Research Laboratory, University of Oxford, OX1 3TA, Oxford, United Kingdom.

^6^ Department of Chemistry, University of Leicester, LE1 7RH, Leicester, United Kingdom.

* These authors contributed equally to this work

^Ψ^ Correspondence:

Alvaro San Millan: Department of Microbiology, Hospital Universitario Ramon y Cajal. Ctra. Colmenar Viejo Km 9,100. 28034, Madrid, Spain.

Email: [alvsanmillan@gmail.com](mailto:alvsanmillan@gmail.com). Phone: +34636737538

Macarena Toll-Riera: Department of Evolutionary Biology and Environmental Studies, University of Zurich, Zurich, CH-8057, Switzerland.

Email: mtollriera@gmail.com. Phone: +41786981582

**Supplementary Material and Methods**

*Bacterial strains, plasmids and culture conditions*

The plasmids used in this study are described in Table 1. *P. aeruginosa* PAO1 was used as recipient strain. To measure plasmid-mediated activation of the SOS response, we used the reporter strain PAO1 WTp*lex*:lux, which was previously constructed in our laboratory (Torres-Barcelo et al 2015). To measure plasmid-mediated activation of QS system, we used the reporter PAO1 P*lasB*::lux (Popat et al 2012). Bacterial strains were cultured in LB broth at 37°C with continuous shaking (225 rpm) and on LB agar plates at 37°C (Fisher Scientific, NJ, USA). PAO1, WTp*lex*:lux and P*lasB*::lux were transformed by electroporation with the different plasmids as previously described (Choi and Schweizer 2006), using a Gene Pulser apparatus (Bio-Rad). Transformants were selected on LB agar plates containing antibiotics as previously described (San Millan et al 2014a, San Millan et al 2014b).

*Competitive fitness assays*

The fitness of each plasmid-carrying PAO1 clone was determined relative to a PAO1-GFP plasmid-free control strain. The GFP label did not produce a significant reduction in fitness in PAO1 (San Millan et al 2014b). Pre-cultures of the strains were incubated at 37°C with 225 rpm shaking overnight in 3 mL of LB broth. Pre-cultures were diluted 20-fold in 200 μL of fresh LB and incubated in the same conditions in 96-well plates until they reached mid-exponential phase (OD_600_ ≈ 0.5). Cultures of the strains were then mixed at a ratio of approximately 50% clone under study to 50% PAO1-GFP. The exact initial proportions were confirmed via flow cytometry using an Accuri C6 Flow Cytometer Instrument (BD Accuri, San Jose, CA, USA) with the following parameters: flow rate: 66 μL min^-1^, core size: 22 μm, events recorded per sample: 10,000. Mixtures were diluted 400-fold in 200 μL of fresh LB and competed in 96-well plates for 16 hours at 37°C with 225 rpm shaking (~8 generations). The final proportion was again measured by flow cytometry. The fitness of the strain carrying the plasmid(s) relative to the PAO1-GFP strain was determined using the formula (Lenski et al 1991):

$$W_{p+}=ln\left( N{final,}_{p+}/N{initial,}_{p+} \right)/ln\left( N{final,}_{p-}/N{initial,}_{p-} \right)$$

where *W_p+_* is the relative fitness of the plasmid-bearing clone, *N_initial,p+_* and *N_final,p+_* are the numbers of cells of the plasmid-carrying clone before and after the competition, and *N_initial,p-_* and *N_final,p-_* are the numbers of PAO1-GFP cells before and after the competition. As a control, PAO1 and PAO1-GFP were competed in every experiment. We performed six biological replicates for each competition.

*Growth curves in biolog EcoPlates*

To assess bacterial growth in different environments, six biological replicates of PAO1 and three biological replicates of each of the six plasmid-carrying PAO1 clones were cultured in Biolog EcoPlates (Biolog, USA). Each clone was pre-cultured in 3 mL of LB overnight (37°C, 225 rpm), diluted down in M9 (1:1000 dilution), inoculated the EcoPlates and then cultured for 20 hours at 37°C. A Tecan Infinite M200 Pro plate reader was used to perform the growth curve experiments (Tecan Trading AG, Switzerland). Growth rates were estimated using the GrowthRates program (Hall et al 2014).

*SOS induction assay*

To assess plasmid-mediated SOS induction, luminescence production was measured over the growth curves of PAO1 WTp*lex*:lux (Torres-Barcelo et al 2015), the different plasmid-carrying WTp*lex*:lux strains, and a control strain with the plasmid-free WTp*lex*:lux growing in the presence of a sub-inhibitory concentration of the SOS-inducing antibiotic ciprofloxacin (45 μg/L). Each clone was pre-cultured in 3 mL of LB overnight (37°C, 225 rpm) and diluted in fresh LB (1:1000 dilution). Eight biological replicates were performed for each strain and the growth curves and luminescence production were measured using a BioTek Synergy H4 plate reader (BioTek Instruments, UK). The area under the curve of the light production over OD_600_ was measured during the exponential phase of the growth curves (first 7 hours) as a proxy for SOS induction (Figure 4).

*Transcriptomics*

RNA-Seq analysis was performed using the RNA samples from six strains: PAO1 wild-type, PAO1/pAMBL1, PAO1/pAMBL2, PAO1/pBS228, PAO1/pAKD1 and PAO1/RmS149. Samples were processed as previously described (San Millan et al 2015). RNA samples were obtained from two biological replicates (from three technical replicates each) per strain at mid-exponential phase of the growth curve (OD_600_ ≈ 0.4-0.5). Both library preparation (directional paired-end ribodepleted library) and sequencing (Illumina MiSeq) were performed at the Oxford Genomics Centre (Wellcome Trust Centre for Human Genetics at the University of Oxford).

RNA-Seq data were analysed using a pipeline that was developed in-house and previously described (San Millan et al 2015). Briefly, the raw reads were filtered using the NGS QC Toolkit (Patel and Jain 2012). Then, the filtered reads were mapped to our reference *P. aeruginosa* PAO1 genome (NC_002516.2 with the insertion of the phage RGP42 GQ141978.1) and to the reference sequence for the 5 plasmids (pAKD1: JN106164.1, RmS149: NC_007100.1, pBS228: NC_008357.1, PAMBL1: KP873172.1, pAMBL2: KP873171.1) using BWA (Li and Durbin 2010). On average, 48.9 x coverage was obtained; 98.7% of the bases had a Phred quality score of 20 or higher and 79.8% of the genes were covered by at least 5 reads. A PCA plot showed that the two biological replicates of each strain clustered together, discarding batch effects (Supplementary Figure S1). HTSeq was used to estimate gene counts (Anders et al 2015), and DESeq2 (version 1.14.1) to perform differential gene expression analysis (Love et al 2014). The PAO1 clone used for these experiments is a laboratory-adapted clone with a mutation (insertion of an IS element) disrupting the promoter region of the quorum sensing (QS) modulator *lasR* gene. Therefore the genes under the control of LasR were excluded in the transcriptional analysis. To confirm the plasmids used in this study did not alter the expression of *P. aeruginosa* QS system, a PAO1 QS reporter strain was used, which contains a chromosomal *luxCDABE* cassette fusion to the promoter of the *lasB* gene, encoding the QS-dependent protease LasB (PAO1 P*lasB*::lux) (Popat et al 2012) (Supplementary Figure 7).

To compare within a given sample the expression of plasmid and chromosomal genes, TPM (transcript per million) was employed. FPKM (fragments per kilobase million) were computed using DESeq2 (Love et al 2014) and then transformed into TPM as follows:

TPM_i_= (FPKM_i_ / Σ_j_ FPKM_j_) x 10^6^

- 1. *Functional enrichment analysis*

The DAVID online tool was used to test for functional enrichment among the groups of differentially expressed genes (Huang da et al 2009). A given term was considered to be enriched when P< 0.05 after Benjamini correction.

*Codon usage computation*

Codon usage for coding sequences from each plasmid and *P. aeruginosa* PAO1 genome was computed using the *cusp* program inside the EMBOSS package (Rice et al 2000). The Codon Adaptation Index (CAI) was used to assess the similarity between the synonymous codon usage of a reference set of genes and that of the synonymous codon usage of plasmid genes. As a reference set, *P. aeruginosa* PAO1 genes coding for ribosomal proteins were used, as they are highly expressed, and it is known that highly expressed genes have an optimized codon usage (Sharp and Li 1987). To compute the CAI, the CAIcal program was used (Puigbo et al 2008). To correct CAI values for gene expression, normalized TPM values were used; normalized TPM values were calculated by dividing each TPM value by the total number of TPMs of a given sample.

*Biosynthetic cost*

The biosynthetic cost of proteins (~P, activated phosphate) was computed by adding up, for each residue of the protein, the energy cost of each amino acid from precursors under respiratory conditions. Energy cost values were obtained from (Wagner 2005), and from (Akashi and Gojobori 2002), leading to very similar results. The biosynthetic costs were computed both for plasmids and *P. aeruginosa* PAO1 proteins. Correction for gene expression was carried out using the same approach used to correct CAI values.

*Untargeted metabolomics*

Samples for the metabolomic analysis were obtained from five biological replicates (prepared on five independent days) for each of the seven clones: PAO1 ancestral strain, PAO1/pAMBL1, PAO1/pAMBL2, PAO1/pBS228, PAO1/pAKD1, PAO1/RmS149 and PAO1/pNUK73. Each clone was pre-cultured in 3 mL of LB overnight (37°C, 225 rpm), diluted in 3 mL of fresh LB (1:30 dilution) and cultured in the same conditions as the day before until they reached an OD_600_ of approximately 0.5. 2 mL of these cultures were then centrifuged to form pellets (6000 rpm, 5 min.), washed in 1.5 mL of M9, and re-suspended in 5 mL of cold 80% methanol to ensure that the cells were dispersed in the methanol solution (and keeping the samples at -20°C). Each sample was then sonicated (40% amplitude, 100 cycles: 5’’ on 10’’ off), on ice to prevent overheating. 0.5 ml of each sample was then centrifuged at 14,800 rpm for 20 min. at 4°C; 200 μl of the supernatant was then transferred to a new tube and stored at -80°C.

Each sample was analysed using ion exchange chromatography coupled to a Q-Exactive HF Hybrid Quadrupole-Orbitrap mass spectrometer. The ion chromatography system (ICS-5000+) incorporated an electrolytic anion generator (KOH), which was programmed to produce a OH^–^ gradient over 37 min. An inline electrolytic suppressor removed OH^–^ ions and cations from the post-column eluent stream prior to MS analysis (Thermo Scientific Dionex AERS 500). A 10 μL partial loop injection was used for all analyses and the chromatographic separation was performed using a Thermo Scientific Dionex IonPac AS11-HC 2 × 250 mm, 4 μm particle size column with a Dionex Ionpac AG11-HC 4 μm 2x50 guard column inline. The IC flow rate was 0.250 mL/min. The total run time was 37 mins and the hydroxide ion gradient comprised as follows: 0 mins, 0 mM; 1 min, 0 mM; 15 mins, 60 mM; 25 mins, 100 mM; 30 mins, 100 mM; 30.1 mins, 0 mM; 37 mins, 0 mM. Analysis was performed in negative ion mode using a scan-range from *m/z* 60-900 and resolution set to 70,000. The tune file source parameters were set as follows: Sheath gas flow 60 mL/min; Aux gas flow 20 mL/min; Spray voltage 3.6v; Capillary temperature 320^°^C; S-lens RF value 70; Heater temperature 350^°^C. AGC target was set to 1e6v ions and the Max IT value was 250 ms. The column temperature was kept at 30°C throughout the experiment. Full scan and MS/MS data were acquired in continuum mode. We used Progenesis QI (Waters, Elstree, UK) software for data processing. This included alignment of retention times, peak picking by identification of natural abundance isotope peaks, characterising multiple adducts forms and identification of metabolites using retention time and fragmentation patterns from authentic metabolite standards and matching accurate mass and isotope abundances experimentally determined with theoretical values. Identifications were accepted according to the following criteria: <5ppm differences between measured and theoretical mass (based on chemical formula), <30 seconds differences between authentic standard and analyte retention times, isotope peak abundance measurements for analytes were >90% matched to the theoretical value generated from the chemical formula. Where measured, fragmentation patterns which were matched to a least the base peak and two additional peak matches in the MS/MS spectrum to within 6 ppm.

The results for each plasmid-carrying PAO1 combination were compared to the results from parental PAO1 to obtain the fold-change difference of metabolites due to plasmid carriage. For our analysis we used those metabolites, identified and non-identified, which were significant (q-value < 0.05) in at least one of the comparisons.

*Statistical analyses*

All statistical analyses and production of graphics were performed using R (R Core Team, 2014).

*Data availability*

The reads generated in this study have been deposited in the European Nucleotide Archive database with the accession number PRJEB24427 (<http://www.ebi.ac.uk/ena/data/view/PRJEB24427>).

**Supplementary References**

Akashi H, Gojobori T (2002). Metabolic efficiency and amino acid composition in the proteomes of Escherichia coli and Bacillus subtilis. *Proc Natl Acad Sci U S A* **99:** 3695-3700.

Anders S, Pyl PT, Huber W (2015). HTSeq-a Python framework to work with high-throughput sequencing data. *Bioinformatics* **31:** 166-169.

Choi KH, Schweizer HP (2006). mini-Tn7 insertion in bacteria with single attTn7 sites: example Pseudomonas aeruginosa. *Nat Protoc* **1:** 153-161.

Hall BG, Acar H, Nandipati A, Barlow M (2014). Growth Rates Made Easy. *Molecular Biology and Evolution* **31:** 232-238.

Huang da W, Sherman BT, Lempicki RA (2009). Systematic and integrative analysis of large gene lists using DAVID bioinformatics resources. *Nat Protoc* **4:** 44-57.

Lenski RE, Rose MR, Simpson SC, Tadler SC (1991). Long-Term Experimental Evolution in Escherichia coli. I. Adaptation and Divergence During 2,000 Generations. *The American Naturalist* **138:** 1315-1341.

Li H, Durbin R (2010). Fast and accurate long-read alignment with Burrows-Wheeler transform. *Bioinformatics* **26:** 589-595.

Love MI, Huber W, Anders S (2014). Moderated estimation of fold change and dispersion for RNA-seq data with DESeq2. *Genome Biol* **15:** 550.

Patel RK, Jain M (2012). NGS QC Toolkit: a toolkit for quality control of next generation sequencing data. *PLoS One* **7:** e30619.

Popat R, Crusz SA, Messina M, Williams P, West SA, Diggle SP (2012). Quorum-sensing and cheating in bacterial biofilms. *Proc Biol Sci* **279:** 4765-4771.

Puigbo P, Bravo IG, Garcia-Vallve S (2008). CAIcal: a combined set of tools to assess codon usage adaptation. *Biol Direct* **3:** 38.

Rice P, Longden I, Bleasby A (2000). EMBOSS: the European Molecular Biology Open Software Suite. *Trends Genet* **16:** 276-277.

San Millan A, Heilbron K, MacLean RC (2014a). Positive epistasis between co-infecting plasmids promotes plasmid survival in bacterial populations. *ISME J* **8:** 601-612.

San Millan A, Peña-Miller R, Toll-Riera M, Halbert ZV, McLean AR, Cooper BS *et al* (2014b). Positive selection and compensatory adaptation interact to stabilize non-transmissible plasmids. *Nat Commun* **5:** 5208.

San Millan A, Toll-Riera M, Qi Q, MacLean RC (2015). Interactions between horizontally acquired genes create a fitness cost in Pseudomonas aeruginosa. *Nat Commun* **6:** 6845.

Sharp PM, Li WH (1987). The codon Adaptation Index--a measure of directional synonymous codon usage bias, and its potential applications. *Nucleic Acids Res* **15:** 1281-1295.

Torres-Barcelo C, Kojadinovic M, Moxon R, MacLean RC (2015). The SOS response increases bacterial fitness, but not evolvability, under a sublethal dose of antibiotic. *Proc Biol Sci* **282:** 20150885.

Wagner A (2005). Energy constraints on the evolution of gene expression. *Mol Biol Evol* **22:** 1365-1374.
